# Exploring G Protein-Coupled Receptors in Hematological Cancers

**DOI:** 10.1021/acsptsci.4c00473

**Published:** 2024-11-21

**Authors:** Choi Har Tsang, Pawel Kozielewicz

**Affiliations:** Molecular Pharmacology of GPCRs, Department Physiology & Pharmacology, Karolinska Institutet, Biomedicum, 171 65 Stockholm, Sweden

**Keywords:** G protein-coupled receptor, acute lymphoblastic leukemia, hematological cancers, RNA sequencing

## Abstract

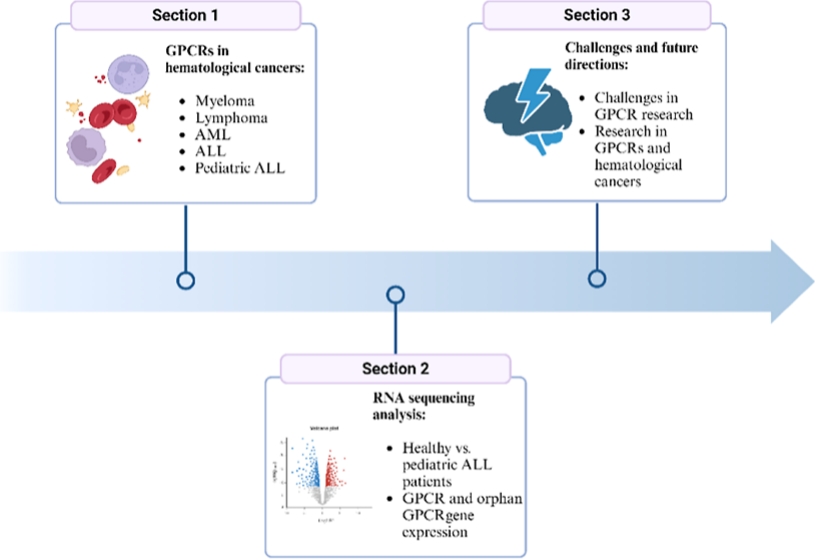

Hematological cancers, such as lymphomas and leukemias, pose significant challenges in oncology, necessitating a deeper understanding of their molecular landscape to enhance therapeutic strategies. This article critically examines and discusses recent research on the roles of G protein-coupled receptors (GPCRs) in myeloma, lymphomas, and leukemias with a particular focus on pediatric acute lymphoblastic (lymphocytic) leukemia (ALL). By utilizing RNA sequencing (RNA-seq), we analyzed GPCR expression patterns in pediatric ALL samples (aged 3–12 years old), with a further focus on Class A orphan GPCRs. Our analysis revealed distinct GPCR expression profiles in pediatric ALL, identifying several candidates with aberrant upregulated expression compared with healthy counterparts. Among these GPCRs, GPR85, GPR65, and GPR183 have varying numbers of studies in the field of hematological cancers and pediatric ALL. Furthermore, we explored missense mutations of pediatric ALL in relation to the RNA gene expression findings, providing insights into the genetic underpinnings of this disease. By integrating both RNA-seq and missense mutation data, this article aims to provide an insightful and broader perspective on the potential correlations between specific GPCR and their roles in pediatric ALL.

## 1

G protein-coupled receptors (GPCRs) constitute the largest superfamily of mammalian transmembrane proteins, including at least 800 seven-transmembrane receptors in humans, which showcases unparalleled diversity.^1^ Each receptor is composed of a single polypeptide chain with a distinct structural layout, where it starts with an extracellular N-terminus and ends with an intracellular C-terminus. These receptors traverse the cell membrane through seven hydrophobic transmembrane domains, connected by three extracellular loops (ECL1, ECL2, and ECL3) and three intracellular loops (ICL1, ICL2, and ICL3).^2^ Among these GPCRs are orphan receptors, a subset of whose endogenous ligands remain unknown. Despite this, orphan GPCRs are believed to play crucial roles in physiological and pathological processes, with some showing high expression in the brain, lymphoid tissues, and the immune system, potentially suggesting relevant roles in biology.^1,3^ Targeting these receptors can modulate a variety of signaling pathways, offering therapeutic potential for a wide range of diseases.^4^ GPCRs have extracellular loops that provide accessible sites for drug binding, while the structural complexity of the transmembrane domains offers opportunities for high specificity, reducing off-target effects and enhancing the efficacy of drug discovery.^4,5^ It is not surprising that they are highly sought-after in modern pharmacology. GPCRs stand as one of the most successful drug targets, with approximately 30% of current pharmaceuticals on the market targeting them.^5,6^

In general, these receptors are essential for translating extracellular signals into diverse intracellular responses, thereby orchestrating numerous physiological processes.^7^ For example, in the blood, adrenergic receptors respond to catecholamines, which are indispensable in the management of cardiovascular conditions and respiratory disorders, with β-blockers and β-agonists serving as therapeutic cornerstones.^8^ Additionally, cannabinoid receptors [cannabinoid receptor 1 (CB1) and cannabinoid receptor 2 (CB2)] are GPCRs, abundantly expressed in the brain, which play a crucial role in modulating pain and appetite.^9,10^ CB1 and CB2 antagonists are used to alleviate chemotherapy-induced nausea, neurodegenerative disorders, and pain.^9−11^

These wide-ranging physiological roles and significant therapeutic potential of GPCRs highlight their importance as pharmacological targets. This is especially pertinent in the context of hematological cancer, where specific GPCRs can critically influence the onset of the disease and its progression. Thus, this study aims to explore GPCRs and their potential roles in various types of hematological cancers.

## 2 GPCRs in Hematological Cancers

Hematological cancers encompass a range of diseases affecting different components of the blood, each affecting the patient differently. This section explores the role of GPCRs in various hematological cancers.

### 2.1 Myeloma

Myeloma, also known as multiple myeloma (MM), is a type of blood cancer that originates in the plasma cells of bone marrow. Plasma cells are a crucial component of the immune system, responsible for producing antibodies.^12^ In myeloma, these cells become cancerous and proliferate uncontrollably, leading to the accumulation of abnormal plasma cells in the bone marrow, resulting in anemia, increased susceptibility to infections, and impaired blood clotting.^12,13^ Currently, there is a 5-year survival rate of approximately 60%, highlighting the need for continued research and development of new therapies with novel mechanisms to improve patient outcomes and manage this complex malignancy more effectively.^14^ Overall, myeloma affects about 7 out of 100,000 people each year in the United States.^16^

To this end, a recent study has emerged to suggest GPCR class C group 5 member D (GPRC5D) as a significant therapeutic target in the treatment of MM. This receptor is mainly expressed in myeloma cells and has limited expression in normal tissues, which reduces the risk of off-target effects, making it an attractive target for therapy.^14^ Consequently, very recent research has focused on the development and clinical evaluation of various GPRC5D-targeting therapies, including bispecific antibodies such as talquetamab, as well as chimeric antigen receptor T cell (CAR-T) therapies such as MCARH109, OriCAR-017, and BMS-986393.^14,15^ While these GPRC5D-targeting therapies have shown promising efficacy in clinical trials (with a 64% or higher positive response rate), they are not without challenges. Bispecific antibodies can cause dermatologic and oral toxicities, as well as a rare cerebellar event, which have been reported with CAR-T therapies.^14,15^ Despite difficulties, this highlights the potential of GPCRs in myeloma, and further research is warranted within this area.

### 2.2 Lymphoma

**Figure 1:**
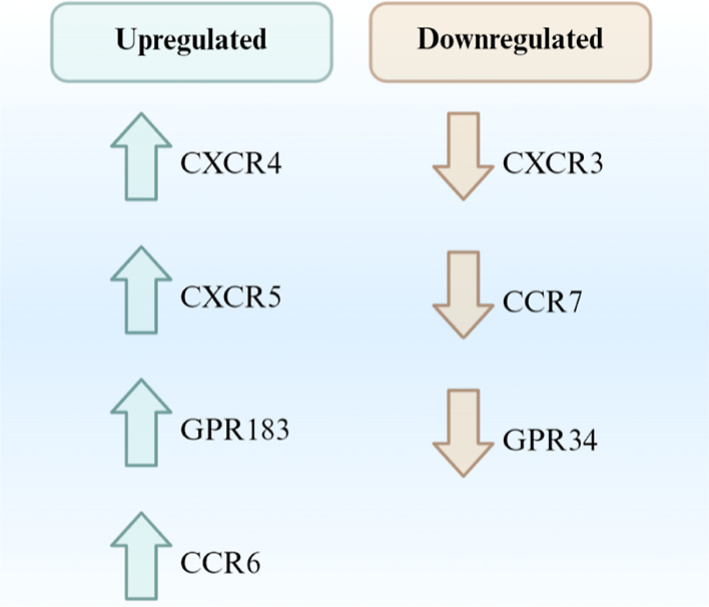
Overview illustrating the dysregulation of a few key GPCRs in lymphomas (created in BioRender).^24,28,51,103−105^

**Figure 2:**
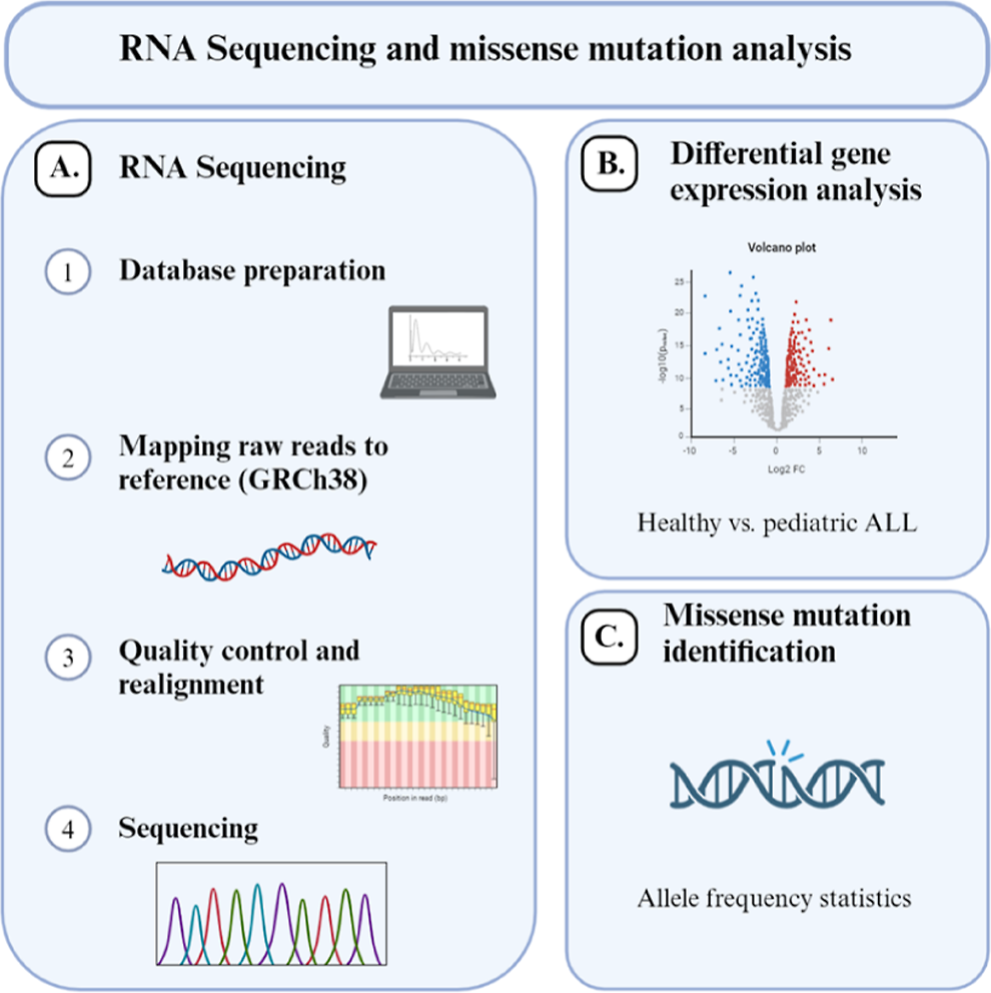
RNA-seq and missense mutation analysis workflow (created in Biorender).

A more prevalent blood cancer in comparison with myeloma is lymphoma, which ranks as the fifth most common cancer in UK adults, and the seventh most common cancer in the United States (including all lymphoma subtypes).^17,18^ Therefore, given the prevalence of lymphomas, it is crucial to explore the roles of GPCRs in this hematological malignancy. Lymphomas manifest as malignant tumors originating within the lymphatic system, an integral component of the body’s immune defenses. The disease emerges when lymphocytes in the lymph nodes or other lymphoid tissues, including the spleen or bone marrow, undergo abnormal proliferation triggered by genetic mutations or other factors, resulting in the formation of cancerous cells, often coalescing into solid tumors within the lymphatic system.^19,20^ In studies, researchers have identified a spectrum of dysregulated GPCRs that play important roles in disease progression. Aberrations in GPCR signaling pathways, such as those involving RhoA, MAPK, and JAK-STAT, can significantly drive the development and advancement of cancer by influencing cellular processes such as proliferation, migration, survival, and apoptosis.^7,21^ These alterations in GPCR signaling pathways may stem from genetic mutations, abnormal expression of GPCRs or their ligands, or changes in downstream signaling molecules.^22,23^ We have crafted a diagram presented in Figure 1 to present a few receptors known for their influence in lymphoma (see Figure 2).

C-X-C motif chemokine receptor 3 (CXCR3) plays a crucial role in mediating immune cell trafficking, particularly the migration of effector T cells to sites of inflammation and tumor microenvironments.^24^ In lymphoma, it has been observed that the downregulation of CXCR3 is a significant molecular event that contributes to tumor progression and immune evasion.^25^ Loss of CXCR3 expression on malignant lymphocytes impairs their ability to respond to CXCR3 ligands such as CXCL9, CXCL10, and CXCL11, which are critical for mediating antitumor effects by directing T cells and NK cells in tumors.^25−27^ This downregulation diminishes T-cell infiltration into the tumor, thereby weakening the host’s immune surveillance and allowing the lymphoma cells to proliferate.^27^ Moreover, the reduced expression of CXCR3 disrupts the local chemokine signaling, further skewing the tumor microenvironment toward immunosuppression.^27^

In contrast, upregulated CXCR4 (C-X-C motif chemokine receptor type 4) is a GPCR that plays a significant role in various physiological and pathological processes.^28^ Publications have suggested CXCR4 signaling to have a significant impact on cell proliferation and leukocyte trafficking in lymphoid organs.^29^ Activation of CXCR4 triggers a cascade of molecular events that upregulate genes essential for cell cycle progression, fostering the expansion of lymphoma cell populations within lymphoid tissues and other disease sites.^30^ This upregulation promotes the transition of cells through various phases of the cell cycle, thereby increasing the overall proliferation rate of lymphoma cells.^30^

In addition to promoting cell proliferation, CXCR4 activation confers a survival advantage by engaging antiapoptotic pathways. Upon binding to its ligand CXCL12, the chemokine receptor CXCR4 initiates downstream signaling cascades involving critical pathways such as PI3K-Akt and MAPK through the activation of AKT and ERK. Subsequently, this activation leads to the suppression of apoptotic responses.^21,31^ This molecular mechanism allows lymphoma cells to evade programmed cell death, thereby contributing to the persistence, survival, and progression of the cancer.^21,31^ As a mediator, plerixafor (marketed as Mozobil), a small-molecule inhibitor, can effectively disrupt the interaction between CXCR4 and its ligand CXCL12. By interfering with CXCR4-mediated signaling pathways, plerixafor impedes the crucial processes of survival, homing, and retention of lymphoma cells within the bone marrow and lymphoid tissues, though this is not directly used as a lymphoma treatment.^32^ This drug is commonly used to mobilize hematopoietic stem cells (HSCs) in non-Hodgkin’s lymphoma (NHL) or MM patients before undergoing stem cell transplantation.^33^ Overall, CXCR4 has been studied under solid tumors and hematological malignancies (such as pancreatic ductal adenocarcinoma and lymphoma).^34−36^ Some studies show that within the microenvironment under solid tumors, interactions with chemokines including CXCL4 can direct the migration of T cells and NK cells, thus promoting antitumor immune cells.^34,37^ These prospects offer promising openings for other potential GPCRs and shed light on the importance of continuous research of these receptors under solid tumors and hematological malignancies.

The above is just a small showcase of how GPCRs play a crucial role in shaping disease progression in lymphoma and myeloma. In general, understanding these dynamics can provide insight into potential therapeutic targets and aid in drug discovery for these patients.

### 2.3 Acute Myeloid Leukemia

Acute myeloid leukemia (AML) is a heterogeneous and aggressive hematologic malignancy characterized by clonal expansion of myeloid progenitor cells in the bone marrow. This uncontrolled proliferation leads to the accumulation of immature myeloid blasts, which disrupt normal hematopoiesis, resulting in anemia, thrombocytopenia, and neutropenia.^38^ Though it is less common in children, it does exhibit dysregulated GPCR expression profiles and accounts for approximately 15–20% of childhood leukemia cases globally.^39^

Recently, G protein-coupled receptor 84 (GPR84) has emerged as a potentially important player in the context of AML.^40^ While its precise role in AML pathogenesis is still being studied, evidence suggests that GPR84 may be crucial in promoting leukemic cell survival, proliferation, and chemoresistance.^40^ The activation of GPR84 is thought to influence the expression of genes associated with cellular processes such as cell cycle regulation, apoptosis, and inflammation. With some studies identifying GPR84 as a new regulator of Catenin beta-1 (β-catenin) that could potentially be a therapy target for AML patients.^40^ However, the exact mechanisms and roles of GPR84 in AML development remains unclear. In general, β-catenin (beta-catenin) has been shown to play a role in the formation of leukemic stem cells (LSCs) in AML.^40,41^ However, directly targeting β-catenin for therapy is challenging due to the lack of effective pharmacological interventions.^40,41^ This study further found that GPR84 is highly expressed in LSCs compared to normal HSCs. However, the suppression of GPR84 expression led to reduced cell growth by inducing G1-phase cell-cycle arrest in preleukemic stem cells (pre-LSCs), which in turn decreased the frequency of LSCs and impaired the reconstitution of AML in mouse and human models.^40^ Overall, this highlights GPR84 as a potential contributor to AML progression.

In addition to GPR84, another G protein-coupled receptor 56 (GPR56, also known as ADGRG1) has been implicated in AML primarily through its interactions with the extracellular matrix. By binding to components such as collagen III and tissue transglutaminase 2, GPR56 facilitates AML cell adhesion to the bone marrow stroma, creating a niche that supports leukemic cell survival and proliferation by activating downstream signaling pathways, such as the RhoA pathway, regulatory of cytoskeletal dynamics and cell migration.^42^

Interestingly, decreased expression of GPR56 has been suggested to be associated with a more aggressive AML phenotype and poorer prognosis.^42,43^ However, the reasons behind this remain unclear; it does shed light on the importance of understanding the dysregulated expression of GPR56 in AML.

By examination of a few receptors, it becomes clear that GPCRs are understudied within AML, despite emerging as important players in AML pathogenesis. This highlights the importance of how a deeper exploration into this area could unveil a deeper understanding of AML, potentially offering novel therapeutic strategies.

### 2.4 Acute Lymphocytic Leukemia

Acute lymphocytic leukemia (ALL) is the most prevalent form of childhood leukemia, accounting for approximately 80% of all childhood leukemia cases globally and it primarily affects children between the ages of 2 and 5 years old.^39,44^ Approximately, 4 of every 10 cases of ALL are in adults, with a 50% rate of relapse and a 65% rate of survival for 5 years or more after diagnosis.^45−47^ ALL constitutes a malignant clonal disorder impacting the hematopoietic system, particularly the blood and bone marrow compartments. Its pathogenesis is characterized by the uncontrolled proliferation of early lymphoid precursors, leading to the replacement of normal hematopoietic elements by proliferating immature lymphoid cells, commonly known as lymphoblasts.^48^

A well-described GPCR in ALL is C–C chemokine receptor type 7 (CCR7), and it had emerged as a significant factor in the pathogenesis and specifically the progression of T-cell ALL (T-ALL).^49^ CCR7 has been implied to play a vital role in the directed migration and lymph node homeostasis through its interaction between the chemokines CCL19 and CCL21.^50^ In T-ALL and lymphocytic leukemia, CCR7 is frequently overexpressed, facilitating the trafficking of leukemic cells to lymphoid organs, where chemokines are abundant. These receptor–chemokine interactions can activate several key pathways, including the PI3K-Akt pathway, which promotes cell survival and proliferation, as well as the MAPK/ERK pathway, which supports cell proliferation and differentiation.^49,51,52^ These mechanisms could lead to cancer cell survival, by suppressing the immune response, and enhance B-cell homing to lymph nodes.^53^ Additionally, CCR7 interacts with CCL19 and CCL21 to activate the Rho guanosine triphosphatase pathway, which regulates cytoskeletal dynamics and cell migration.^53^ Collectively, these processes allow T-ALL cells to exploit protective niches, driving chemotherapy resistance and ALL disease persistence.

In addition to CCR7, a lesser ALL-characterized GPCR called G protein-coupled receptor 183 (GPR183) (also known as EBI2 (Epstein–Barr virus-induced molecule 2) has recently gathered some attention in the context of lymphoma and ALL. Initially recognized for its role in immune responses and inflammation, emerging evidence suggests potential implications of GPR183 dysregulation in leukemic pathogenesis.^54^ This is further supported by the fact that GPR183 agonist, 7α,25-dihydroxycholesterol (7α,25-OHC), has been linked to direct cell migration of B cells and T cells, both known to impact ALL progression.^55^

Moreover, our RNA sequencing (RNA-seq) data (found in Integration of Expression Data and Clinical Relevance) provide preliminary evidence of the dysregulation of GPR183, suggesting a potential role in pediatric ALL. However, research of GPR183 involved in ALL is relatively limited, and more comprehensive studies are needed to understand the molecular mechanisms underlying GPR183 actions.

#### 2.4.1 Pediatric ALL

As mentioned previously, pediatric leukemia is one of the most common cancers in children, especially impacting those under 5 years old.^56^ There are two types of childhood leukemias: ALL and AML. However, pediatric ALL is most prevalent out of the two, accounting for approximately 3 out of 4 leukemia cases.^39^ It also tends to be more aggressive compared to adult ALL and is frequently associated with high-risk genetic abnormalities, such as the Philadelphia chromosome (Ph + ALL) and mixed lineage leukemia rearrangements.^57^ Furthermore, pediatric cancer survivors face different long-term health challenges compared to adults, making it essential to develop treatments that minimize adverse effects and improve quality of life.^44^ Given the current limited research on childhood ALL, focusing on this area can fill critical knowledge gaps and lead to innovative treatments tailored specifically for children.

As mentioned throughout, GPCRs respond to a variety of extracellular stimuli, including hormones, neurotransmitters, and chemokines, thereby influencing numerous physiological processes, with emerging evidence suggesting dysregulated expression and signaling of GPCRs, such as GPR84, CXCR4, CXCR5, and others, to play pivotal roles within hematological cancers; it is important to further explore the role of GPCRs in childhood ALL.^39,58^ Thereby, we utilized existing raw RNA-seq data (preliminary results can be found in “Integration of Expression Data and Clinical Relevance”) to highlight the importance of up-and-down regulation in understanding the involvement of GPCRs in childhood ALL and shed some light on the molecular mechanisms underlying this disease.

## 3 Integration of Expression Data and Clinical Relevance

Leveraging existing data, particularly in underresearched areas such as pediatric ALL, provides valuable insights into the complexities of diseases. In this study, we have analyzed raw RNA-seq data sets sourced from pediatric bone marrow donors, available in the NCBI’s Gene Expression Omnibus under accession numbers GSM4664009 (healthy samples) and GSE109807 (ALL samples, aged 3–12 years).

**Figure 3:**
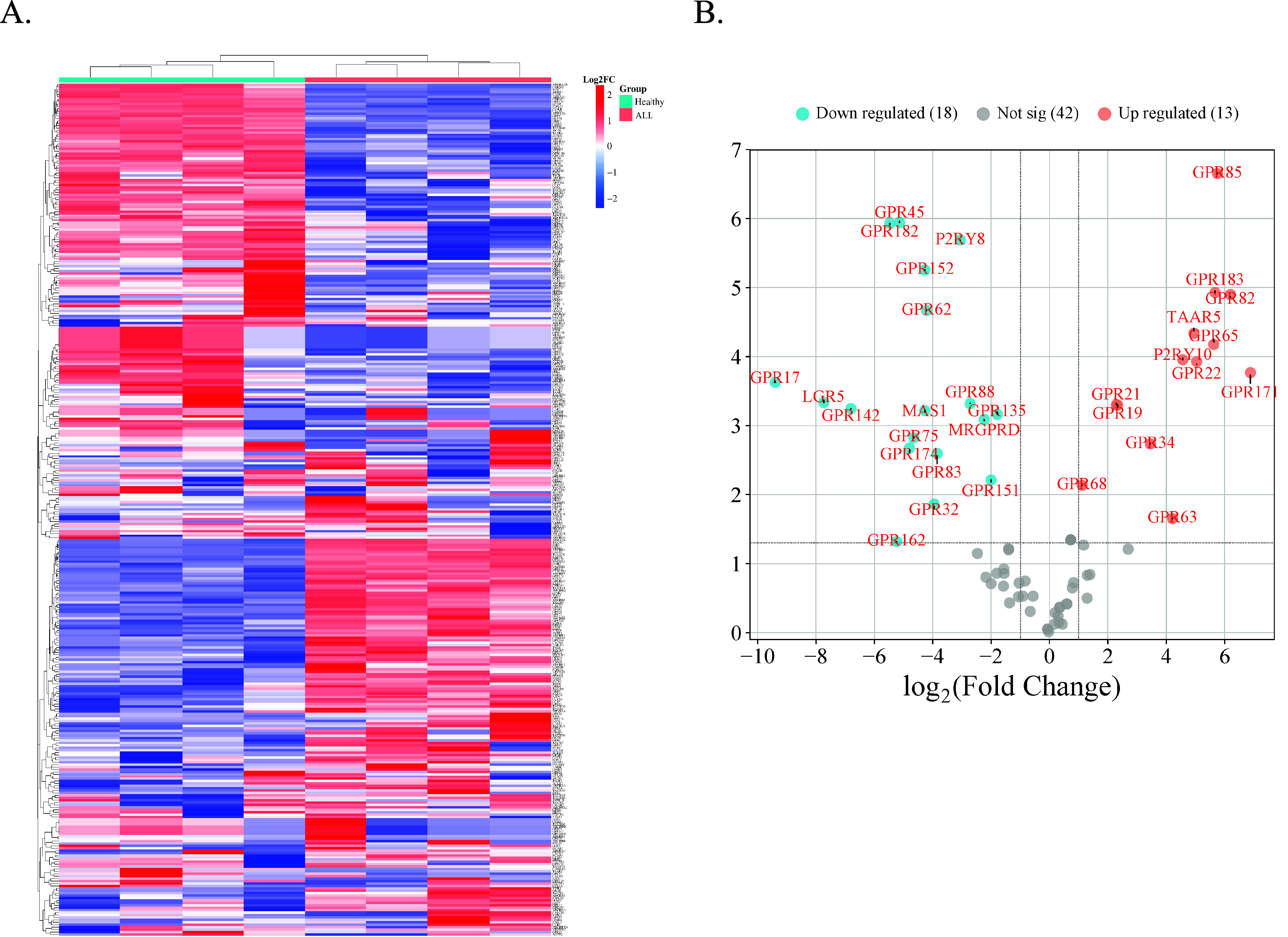
(A) Heatmap representing all differentially expressed GPCRs across all samples. Comparison between ALL patients and healthy controls (*p* value < 0.05). (B) Volcano plot illustrating significantly expressed orphan GPCRs, based on log2 fold change (log2 FC) ratios between samples from healthy children and those with ALL (*p*-value < 0.05).

Our analysis revealed distinctive GPCR expression profiles in childhood ALL, identifying several candidates such as GPR183, GPR85, and GPR82, which exhibited aberrantly upregulated expression compared to healthy counterparts. In contrast, G protein-coupled receptor 45 (GPR45), P2RY8, and G protein-coupled receptor 182 (GPR182) were notably downregulated in ALL samples (Figure 3).

### 3.1 Methods

The raw Illumina RNA-seq data sets with NCBI’s Gene Expression Omnibus accession numbers GSM4664009 (healthy samples) and GSE109807 (ALL samples, aged 3–12 years) were mapped against human (GRCh38) with BWA. After quality checks and duplicate removal, all reads were normalized using Feature count. Differential expression analysis was carried out in Rstudio using the Limma-voom *t*-test (*p* value < 0.05).^59^ The analysis focused on GPCRs, filtering for Class A, B, C and Frizzleds, with separate filtration for Class A orphan GPCRs. Missense mutations in differentially expressed genes were analyzed, concentrating on significantly up- and downregulated orphan GPCRs in pediatric ALL. The Catalogue of Somatic Mutations in Cancer database was used to identify relevant mutations, excluding genes not found in pediatric ALL.^60^ These mutations were cross-referenced with the GnomAD database to obtain naturally occurring allele frequency statistics.^61^

### 3.2 Results and Discussion

According to Figure 3B, we found 13 significantly upregulated (including GPR85, GPR183, and GPR82) and 18 significantly downregulated (such as GPR45, GPR182, and P2RY8) orphan GPCRs.

G protein-coupled receptor 85 (GPR85) was found to be the most upregulated gene (Figure 3B), though this receptor is mainly expressed in the brain and contributes to various neurological diseases such as schizophrenia; there is limited research correlating this receptor to hematological cancers. Interestingly, a study revealed GPR85 as a novel functional receptor for CXCL14 activity.^62,63^ CXCL14, described as a homeostatic chemokine, recruits macrophages playing a crucial role in enhancing macrophage infiltration as well as proliferation and migration.^64^ Macrophages are also known to participate in leukemia progression, by secreting cytokines and growth factors.^65,66^ Potentially, GPR85 may not directly be involved in ALL progression but can serve as a communicator to other receptors driving the development of ALL. However, further research is warranted.

The second most upregulated gene, G protein-coupled receptor 183 (GPR183), has been already mentioned in this article. This receptor has been suggested to play a key role in the migration of B cells and T cells via 7α,25-OHC.^67^ A recent study further suggests that GPR183 is a critical regulator of immune responses while being a potential prognostic marker and a therapeutic target in AML. Next, it has been demonstrated that elevated GPR183 expression correlates with poor prognosis in AML, regardless of age and treatment response.^54^ Knockdown (KD) of GPR183 using specific short hairpin RNA in AML cells inhibited proliferation, promoted apoptosis, and reduced tumor burden in vivo, suggesting therapeutic potential.^54^ Furthermore, GPR183 overexpression exacerbated AML growth and drug resistance. These findings emphasize the significance of GPR183 in AML pathogenesis and highlight its potential as a candidate for targeted therapy.

GPR82, a receptor that has not been explored within blood cancers but commonly investigated under metabolic diseases thus far, is expressed in lymphoid tissues and bone-marrow-derived dendritic cells.^68^ A very recent study (2023) suggested a potential ligand for GPR82 called Lysophosphatidylserine.^69^ LysoPS can modulate T cell function by suppressing interleukin-2 production in CD4 T cells, which could impact the progression of blood cancers, but this requires more research. This study also found GPR82 to inhibit G_i_ protein activation, while an artificial lysophospholipid that exerted antitumor activity was present.^69^ G_i_ protein activation involves the inhibition of the cAMP-dependent pathway, which can promote tumor developing properties. Furthermore, the dysregulation of the cAMP-dependent pathway can be seen in leukemia patients.^70,71^ It seems GPR82 could have an impact within hematological cancers, but further investigation is needed.

Moving on to downregulated genes, a few include GPR45, GPR182, and P2RY8. First, G protein-coupled receptor 45 (GPR45), to our knowledge, has not been studied in the context of hematological malignancies. However, knockout studies showed GPR45 to have a correlation with glucose intolerance, causing a higher level of glucose in the bloodstream.^72^ This is important as further studies have shown that high levels of glucose lead to leukemia cell growth and progression, particularly in AML where it is also correlated with a 40% higher death rate.^73^ Notably, research into the roles of GPR45 in AML and ALL should be of interest in the future.

G protein-coupled receptor 182 (GPR182), although a classified orphan, has some well-described ligands. For instance, a study using GPR182 KD mice presented potential binding to CXCL10 and CXCL12.^74^ During KD, both chemokines were present in higher levels.^74^ First, CXCL10 is a chemokine ligand that is expressed primarily in lymphoid tissues and regulates immune responses through the activation and recruitment of leukocytes such as monocytes and T cells.

Another potential binding ligand named CXCL12 is a homeostatic chemokine, and it activates or stimulates the migration of hematopoietic progenitor and different leukocytes.^75^ According to a study, a higher expression of CXCL12 has been correlated to pediatric ALL patients.^76,77^ Therefore, downregulation of GPR182 could potentially impact the progression of this disease. Furthermore, this receptor is highly constitutive in β-arrestin recruitment.^74^ The progression and onset of leukemia are β-arrestin dependent, which has been seen in myeloid leukemia patients with β-arrestin2.^78^ Due to this, the dysregulation of β-arrestin can impact on the development of leukemogenesis; for example, a study found significantly elevated levels of β-arrestin1 in 155 ALL patients.^79^ However, the roles of β-arrestin1 and β-arrestin2 in leukemia are unclear. Overall, GPR182 could provide promising avenues for developing therapeutic strategies for hematological cancers.

Next, P2RY8 is a purinergic receptor that has been characterized to promote B-cell development and migration while being highly expressed in lymphocytes.^80^ In support of our findings, a study found P2RY8 to be downregulated during leucocyte differentiation and undifferentiated promyelocytic leukemia cell line to also have a downregulation of P2RY8.^81^

Furthermore, CRLF2-P2RY8 fusion genes are usually linked together in hematological cancer. It is believed that CRLF2 can be translocated to the P2RY8 locus on chromosome X (Xq26.3), which can occur through a deletion in the pseudoautosomal region or mutation, creating a fusion that may contribute to the oncogenic processes and development.^82^ This highlights the importance of mutational profiling within GPCRs and hematological cancers.

To gain a deeper insight into the genetic landscapes of the orphan GPCRs found from the RNA-seq, we conducted further analysis in the exploration of missense mutations in relation to the differentially expressed genes identified in Figure 3B. For this analysis, we used somatic cancer data as these mutations are not inherited but instead involve genetic changes that occur in specific cells during a person’s lifetime. Although somatic cancer data consider hereditary predispositions only, we believe somatic data are more suitable for exploring a wider range and providing more insight into our findings.

It is important to note, gene expression is the process by which information from a gene is used to synthesize functional gene products, which can be profoundly impacted by mutations such as missense mutations.^83^ It involves a single nucleotide change resulting in the substitution of one amino acid for another within the protein sequence.^84^ Depending on the location and nature of the substitution, missense mutations can alter the protein’s structure, stability, or function, causing a dysregulation of gene expression.^83^ However, not all cause a loss of function as some can lead to significant changes in protein function and expression, which can impact their roles in hematological cancers.^84^ Therefore, we wanted to provide some insight into the potential interplay between gene expression and missense mutations of orphan GPCRs found in Figure 3B.

**Figure 4:**
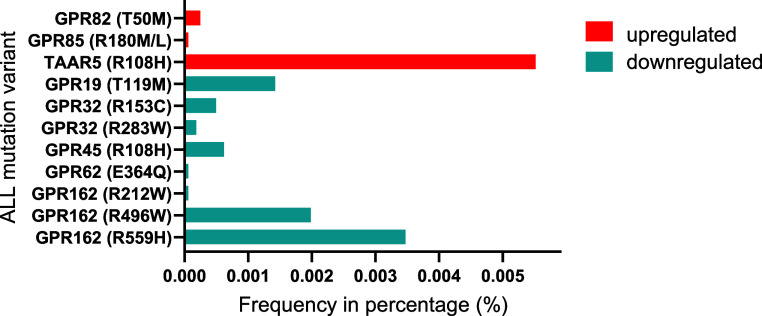
Naturally occurring allele frequencies of missense mutations in pediatric ALL are shown in relation to the differentially expressed genes found in Figure 3B. Missense mutations were identified separately using the COSMI IC database, which are indicated in brackets within the graph.^60,61^

As presented in Figure 4, we identified 11 different ALL missense mutation variants. G protein-coupled receptor 162 (GPR162) presented the most variants, with G protein-coupled receptor 32 (GPR32) being second. Both were found to be downregulated in our findings.

Our findings revealed GPR162 to have three different ALL mutational variants including positions R559H, R496W, and R212W (Figure 4). While research into GPR162 is quite limited, a study did find GPR162 to play some role in tumor suppression by activating the STING-dependent DNA damage pathway, thereby acting as a novel tumor suppressor while promoting the expression of CXCL10.^85^ As mentioned previously, CXCL10 has a role in leukocytes recruitment and potentially could be antileukemic.^74^ Moreover, further studies indicated the STING-dependent DNA damage pathway to mediate antitumor immunity and inflammatory responses.^86^ In general, GPR162 could have an interesting role in the context of cancer but certainly needs further investigation.

On the other hand, GPR32 has a putative agonist called resolvin D1 (RvD1) with high affinity binding, which has been connected to regulate leukocyte responses, including enhancing macrophage phagocytosis.^87^ RvD1 further has been correlated with anti-inflammatory responses as well as decreasing cancer growth by stimulating monocytes.^88^ In this case, the downregulation of GPR32 could potentially have some crucial roles in ALL. Moreover, mutational changes such as the ones we found (R153C and R283W (Figure 4)) can significantly impact the binding of RvD1 to the protein. In some cases, structural alterations in the protein may prevent RvD1 from binding at all. Therefore, further research is needed in the areas of mutational profiling and ligand discovery, particularly in the context of ALL research.

Again, we stress that this analysis is to provide an insight into the complexity of this disease. The identification of missense mutations can determine the ligand binding potential and the roles of a receptor. Though in conclusion, our integration of RNA-seq and missense mutation data reveal critical molecular dysregulation in pediatric ALL, particularly that involving orphan GPCRs. This approach enhances our understanding of leukemogenesis and identifies potential biomarkers.

## 4 Challenges and Future Directions

Investigating GPCRs in blood cancers presents significant challenges due to the complex structure of these receptors and their intricate signaling pathways. The GPCR family, comprising over 800 members, exhibit remarkable structural diversity, with each subtype possessing unique conformational states and ligand-binding sites.^89^ Additionally, the identification and optimization of ligands that can selectively target specific GPCRs involved in leukemogenesis are hindered by the dynamic and flexible nature of these receptors, often leading to promiscuous binding and off-target effects.^90^

As demonstrated within this work, leveraging advanced tools such as RNA-seq can correlate GPCR activity with disease biology, enabling the identification of key players that can potentially be therapeutic targets. However, RNA-seq alone does not provide the whole picture. While RNA-seq offers valuable insights into transcriptomic profiles, it lacks the ability to fully capture post-transcriptional modifications, protein abundance, and functional states of proteins. To address these limitations, integrating RNA-seq data with other omics approaches, such as proteomics and epigenomics, can provide a more comprehensive view of gene expression and regulation.^91,92^ Proteomics complements RNA-seq by quantifying protein levels and modifications, thereby linking transcriptomic changes to functional protein outcomes.^91^ Similarly, epigenomics can reveal regulatory mechanisms controlling gene expression, such as DNA methylation and histone modifications, offering deeper insights into transcriptional regulation.^92^ This multiomics integration facilitates a more holistic understanding of biological processes, enhancing the identification of robust therapeutic targets and biomarkers. Furthermore, implicating precise gene-editing techniques, such as CRISPR-Cas9, can facilitate functional studies of GPCRs in a relevant biological context.^93^ Collaborative efforts integrating clinical data with cutting-edge research methodologies are crucial for overcoming hurdles and translating GPCR-targeted therapies into clinical practice, ultimately enhancing the management and outcomes of lymphomas and childhood leukemia.

GPCRs are exemplary drug targets due to their ubiquitous expression across nearly all tissues and cell types, making them relevant to a multitude of diseases and therapeutic areas, including cardiovascular, respiratory, and metabolic disorders and cancer. Currently, there are only eight FDA-approved drug targets for GPCRs in cancer therapy.^94^ Structurally, GPCRs possess well-defined binding pockets including orthosteric binding pockets that facilitate the design of small molecules, peptides, and antibodies that can specifically alter receptors’ activity.^95^ Moreover, the presence of allosteric sites on GPCRs offers opportunities for developing drugs with novel mechanisms of action, allowing for fine-tuning of receptor activity with potentially improved pharmacological interventions and efficacy profiles.^96,97^

Additionally, GPCRs exhibit high conformational flexibility, adopting multiple states during activation and signaling, which complicates the capturing of these transient states with traditional methods.^90^ To overcome the structural challenges inherent in studying GPCRs, advanced techniques such as cryoelectron microscopy (cryo-EM) and computational modeling are necessary. Cryo-EM can address these challenges by providing high-resolution images of GPCRs in different conformational states, enabling detailed visualization of receptor–ligand interactions.^98,99^ Computational modeling and molecular dynamic simulations can complement this by simulating dynamic behaviors and predicting interactions that are challenging to capture experimentally.^100,101^ Furthermore, techniques such as high-throughput screening (HTS) and structure-based drug design are also vital as they facilitate the identification and optimization of selective ligands for a target through the use of large databases, which are crucial for targeting specific GPCR subtypes with minimal off-target effects.^102^ Collectively, combining structural, computational, and omics technologies provides a robust framework for advancing our understanding of GPCR biology and accelerating the discovery of novel therapeutic targets.

## 5 Conclusions

Advanced tools such as RNA-seq provided valuable insight into the dysregulations of orphan GPCRs including 13 upregulated (including GPR85, GPR183, and GPR82) and 18 significantly downregulated (such as GPR45, GPR182, and P2RY8) within childhood ALL patients. However, this amount of data is insufficient alone for a deeper understanding into these orphan GPCRs, and integrating RNA-seq with others offers a more comprehensive understanding of gene expression and regulation. As mentioned previously, complementary techniques like cryo-EM and computational modeling provide detailed structural insights, while HTS and structure-based drug design aid in identifying selective ligands. Additionally, precise gene-editing techniques such as CRISPR-Cas9 can be important for functional studies of GPCRs in relevant biological contexts. Collaborative efforts that integrate clinical data with cutting-edge research methodologies are pivotal for overcoming the hurdles in GPCR-targeted therapy development. This integrative approach will not only enhance our understanding of GPCR biology in lymphomas and leukemias but will also accelerate the discovery of novel therapeutic targets, ultimately improving the management and outcomes of these patients. However, there remains a significant gap in our knowledge. It is evident that a critical investigation of GPCR expression in pediatric leukemias and lymphomas is essential to highlight the importance of key receptors in the development and progression of these diseases. Continued research is imperative to fully understand the roles of GPCRs and translate these insights into effective therapeutic strategies. Ultimately, collaborative efforts in research and methodology can aid in overcoming the challenges in treating pediatric blood cancers and significantly enhance the prognosis and quality of life for affected children as well as adults.
